# Pneumonia Classification from X-ray Images with Inception-V3 and Convolutional Neural Network

**DOI:** 10.3390/diagnostics12051280

**Published:** 2022-05-21

**Authors:** Muhammad Mujahid, Furqan Rustam, Roberto Álvarez, Juan Luis Vidal Mazón, Isabel de la Torre Díez, Imran Ashraf

**Affiliations:** 1 Department of Computer Science, Khwaja Fareed University of Engineering and Information Technology, Rahim Yar Khan 64200, Pakistan; mujahidws890@gmail.com; 2 Department of Software Engineering, University of Management and Technology, Lahore 54770, Pakistan; furqan.rustam1@gmail.com; 3 Higher Polytechnic School/Industrial Organization Engineering, Universidad Europea del Atlántico, Parque Científico y Tecnológico de Cantabria, C/Isabel Torres 21, 39011 Santander, Spain; roberto.alvarez@uneatlantico.es (R.Á.); juanluis.vidal@uneatlantico.es (J.L.V.M.); 4 Department of Project Management, Universidad Internacional Iberoamericana, Campeche 24560, Mexico; 5 Project Department, Universidade Internacional do Cuanza Bairro Kaluanda, Cuito EN 250, Bié, Angola; 6 Department of Signal Theory and Communications and Telematic Engineering, University of Valladolid, Paseo de Belén 15, 47011 Valladolid, Spain; 7 Department of Information and Communication Engineering, Yeungnam University, Gyeongsan 38541, Korea

**Keywords:** pneumonia, chest X-ray, ensemble learning, deep learning

## Abstract

Pneumonia is one of the leading causes of death in both infants and elderly people, with approximately 4 million deaths each year. It may be a virus, bacterial, or fungal, depending on the contagious pathogen that damages the lung’s tiny air sacs (alveoli). Patients with underlying disorders such as asthma, a weakened immune system, hospitalized babies, and older persons on ventilators are all at risk, particularly if pneumonia is not detected early. Despite the existing approaches for its diagnosis, low accuracy and efficiency require further research for more accurate systems. This study is a similar endeavor for the detection of pneumonia by the use of X-ray images. The dataset is preprocessed to make it suitable for transfer learning tasks. Different pre-trained convolutional neural network (CNN) variants are utilized, including VGG16, Inception-v3, and ResNet50. Ensembles are made by incorporating CNN with Inception-V3, VGG-16, and ResNet50. Besides the common evaluation metrics, the performance of the pre-trained and ensemble deep learning models is measured with Cohen’s kappa as well as the area under the curve (AUC). Experimental results show that Inception-V3 with CNN attained the highest accuracy and recall score of 99.29% and 99.73%, respectively.

## 1. Introduction

Pneumonia has been regarded as a leading cause of death, with 14% of deaths for children under the age of 5 and a reported 740,180 deaths among children in 2019 [1]. Symptoms include a vigorous or dry cough, a high-grade fever with cramps, rapid breathing, shortness of breath, chest discomfort when coughing, a fast heartbeat, feeling extremely fatigued or weak, nausea and vomiting, diarrhea, seizures, etc. Patients with severe symptoms experience loss of appetite, bodily discomfort, and coughing up blood or cyanosis. Pneumonia can be transmitted in a variety of ways. When a child breathes, viruses and bacteria often present in their nose or throat might infect the lungs. When they cough or sneeze, it can be spread by airborne droplets. Globally, millions of individuals are affected by pneumonia every year. Overall, it is the third major cause of death. In 2016, 860,373 children under the age of five died from pneumonia, while 808,694 died in 2017. Every year, 90,000 children in Pakistan die from pneumonia. Even though it is more frequent in South Asia and Sub Saharan-Africa, pneumonia affects families and children all over the world. Children under the age of five are highly susceptible to pneumonia in countries with high mortality rates, and in Pakistan, it is the second leading cause of death for such children. Pakistan’s Abbottabad City had a mortality rate from pneumonia of 14 deaths for every 1000 children under the age of 5 prior to interventions. In a hamlet in Pakistan’s Northern Areas, pneumonia killed 44 percent of the children under the age of five between 1988 and 1991, according to formal autopsy techniques. For infants and children aged 1 to 4, pneumonia remains a major cause of death in the Northern Areas, according to a verbal autopsy conducted by the ‘Aga Khan Health Services’, Pakistan [2,3,4].

Pneumonia is more frequent in poor and impoverished countries, where overcrowding, pollution, and unsanitary climatic factors predominate. This has aggravated the situation, and medical supplies are short. As a result, early detection and care can make a significant difference in preventing the disease from spreading to a fatal stage. For diagnosis, radiographic evaluation of the lungs by utilizing computed tomography (CT), magnetic resonance imaging (MRI), and radiographs (X-rays) is commonly employed. X-ray radiography is a congenital and generally a low-cost assessment of the lungs [5]. The white patches in the pneumonic X-ray that distinguish a pneumonic state from a healthy one are known as infiltrates. In contrast, chest X-ray diagnostics for pneumonia identification are vulnerable to subjective variability. As a result, an automated approach for detecting pneumonia is necessary.

Over conventional testing techniques, X-ray imaging technology has several benefits as an alternative diagnosis procedure for pneumonia. These advantages incorporate its low operating cost, widespread accessibility for X-ray capabilities, non-invasiveness, reduced computation complexity, and equipment simplicity. Given the present global healthcare crisis, X-ray imaging might be a superior option for the large-scale, simple, inexpensive, and rapid identification of a pandemic, as well as pneumonia, COVID-19, heart failure, bone fracture, and so on [6]. Over the last few years, computer-controlled pneumonia diagnosis systems have become increasingly popular as a way to enhance the efficiency and validity of clinical services. Deep learning approaches go beyond standard machine learning methods for various image analyses in the medical field applications, as in tracking, categorization, and classification [7].

Deep learning is a powerful artificial intelligence technology that can help solve complex computer vision problems [8]. For image categorization tasks, deep learning models, particularly convolutional neural networks (CNN), are frequently used [9,10]. Being data-intensive models, these models require a large amount of data for better performance, which is difficult for biomedical image classification issues since it needs experienced doctors to identify each image. Transfer learning [11] is a workaround to overcome this barrier. In this strategy, a model trained on a large dataset is reused to resolve a problem involving a small dataset, and the network weights calculated in this model are used. In this study, we utilized different pre-trained networks to detect pneumonia in its early stages and make the following contributions.

- An ensemble model is derived where a CNN model is joined with the pre-trained Inception-V3 to obtain accurate results for pneumonia classification, thus investigating the influence of transfer learning for enhancement in models’ performance.
- Data augmentation is used to investigate the impact of artificially increased data on the accuracy of models trained using deep learning. The impact of data augmentation is also studied for model overfitting.
- Performance of models is analyzed regarding the use of single models vs. custom-made ensemble models for the task of pneumonia detection. Additionally, a comparison to state-of-the-art models is also performed.

The rest of this paper is formulated as follows. Section 2 covers the related work for the current study. Preprocessing, data splitting, and deep learning models are all covered in Section 3. The results and discussion are provided in Section 4, while the conclusion is given in Section 5.

## 2. Literature Review

Many researchers have been working in the domain of disease detection to develop automated detection models. Deep learning has already been used to efficiently enhance productivity, based on computer-assisted diagnosis technologies, particularly in the fields of medical imaging, image classification, and image restoration [12,13]. A large number of patients and a shortage of medical experts and supporting staff are the primary difficulties for pneumonia detection. It can be difficult for radiologists to identify pneumonia using chest X-rays. The presence of pneumonia-containing CXR images is rarely unidentified, may be masked by various findings, and can simulate many other harmful irregularities. These inconsistencies cause striking contradictions among radiologists in determining pneumonia. Many research studies have developed different algorithms to detect diseases and save lives. CNN has been used to perform a vast range of classification problems associated with medical diagnosis, including breast cancer detection [14], skin cancer classification [15], interpretation of chest X-ray pictures to identify the disease [16], identification of white blood cell cancer using images of bone marrow [17,18], and more.

The author [19] utilized the new chest X-ray-8 dataset comprising 108,948 chest X-ray images of 32,717 unique patients, gathered from 1992 to 2015, with eight illness image labels and multiple labels on each image. For identifying problematic terms, eliminating negation, and reducing ambiguity, several natural language processing approaches are used. Experiments are performed using deep CNN to discriminate eight distinct thoracic disorders and medical complications. Guan et al. [20] presented a deep learning model, the attention-guided convolutional neural network (AGC-NN), to demonstrate a distinction between the eight malignancies identified in the previous paper. Singh et al. [21] assessed the performance of deep learning methods for identifying abnormalities in regular frontal chest radiographs and the stability or change in findings over time. Another study [22] employed a transfer learning strategy in conjunction with deep CNN to identify pneumonia through chest radiographs.

For classifying pneumonia based on X-ray images, ref. [23] utilized an ensemble learning approach. Cropping and histogram equalization were also used as image preparation methods since cropped images and histogram equalization improve image quality and reduce unnecessary information. Experiments were conducted using the Kaggle dataset, and the study achieved the best accuracy of 97%. Ensemble learning is employed in [24] to derive features from an X-ray to confirm the presence of pneumonia and identify the type of pneumonia. To balance the dataset and achieve the best identification result, an image augmentation approach is also applied. Another study on pneumonia detection is [25], which utilizes a large dataset of 5216 images. An optimized CNN architecture is used to detect viral, bacterial, and fungal infections using the X-ray images. Different architectures are studied for analyzing the influence of the number of layers and other parameters on the training time and obtained accuracy. The ensemble deep learning approach was utilized in the study [26] to detect COVID-19 and pneumonia in a joint dataset. In a similar fashion, ref. [27] used the deep CNN model to detect pneumonia from chest X-ray images. The authors of [28] employed ensemble models for the detection of pneumonia. Similarly, ref. [29] utilized ensemble models for the detection of COVID-19 from X-ray images. Ensemble deep learning is utilized in the study [30] to classify heart disease. The major goal of these studies is to improve the accuracy of classification for imbalanced datasets.

The study [31] employed fine-tuned parameters and information obtained via transfer learning to develop ensemble learning models for COVID-19 identification from chest X-rays. Another similar study is [32], which proposed a CNN-based deep learning model. Images are resized and preprocessed for better results. Different parameters are optimized to reduce model overfit and obtain better results. The authors present a deep learning approach for pneumonia deduction in [33]. The efficacy of the suggested model is improved by the use of several parameter optimization techniques. Ref. [34] used a deep CNN architecture with data augmentation for pneumonia. CNN is studied with different architectures to achieve the best results, and results are analyzed with and without data augmentation. Results suggest that, with the augmentation approach, the accuracy is 83.38%, while with the original dataset, it is only 80.25%. A comparative and analytical summary of the discussed works is given in Table 1.

Results of the aforementioned works indicate that the ensemble models outperform the single models in terms of performance, both for machine learning and deep learning models. Similarly, the results suggest that data augmentation helps to reduce the probability of model overfit and increases performance. The use of transfer learning, where a pre-trained model is used for pneumonia, is reported to have high accuracy. In view of these facts, this study adopts the use of the pre-trained models to generate an ensemble with the CNN model.

## 3. Proposed Methodology

**Figure 1:**
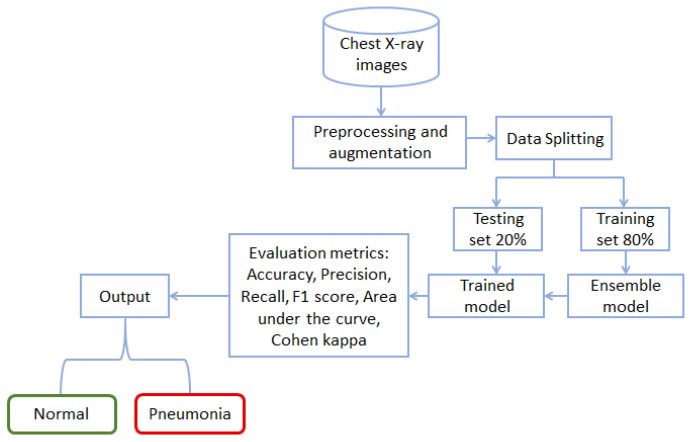
The workflow of the proposed methodology.

This section briefly covers the dataset description, preprocessing, deep learning models, and evaluation metrics. Figure 1 shows the workflow diagram of the methodology followed in this study. Starting with the chest X-ray data, the study follows preprocessing and data augmentation. Preprocessed data are later used to train the proposed model for training and testing. The performance of the trained model is then analyzed using the unseen data, and accuracy and other well-known performance metrics are used.

### 3.1. Dataset Description, Preprocessing, and Augmentation

The pneumonia chest X-ray image dataset for the current study is obtained from Kaggle [35]. The dataset contains 7750 X-ray images in total, where 6200 are used for training while 1550 are used as the test set. For preprocessing, X-ray images are resized. The original images are recorded in different dimensions, such as 1344 × 600, 1272 × 1144, etc. Therefore, from a model implementation and computational perspective, the X-ray images are resized to a standard size. All images are converted into 150 × 150 × 3 size, indicating the height, width, and the number of channels, respectively. The number of channels is three and includes red, green, and blue. After the image resizing, data augmentation is performed to increase the size of the dataset.

For data augmentation, the image augmentor Python library is used to balance the normal and pneumonia class images. The flow of the data augmentation is provided in Algorithm 1. The data augmentation follows a series of sequential steps, starting with the rotation. It is followed by the zoom, flip_left_right, and flip_top_bottom functions. In the end, the random_distortion function and sample rate are used. Data augmentation is applied only to the normal images because the number of pneumonia X-ray images is already high in the original dataset. Normal images are generated to balance the dataset, which reduces the model overfitting for majority class data.
**Algorithm 1** Data augmentation**Input:** Normal class images**Result:**$Aug_{normal}$ dataset ⟶Equal number of normal and pneumonia class images1:**for***i* to *N* images in $CXR_{normal}$ **do**2:   P[i]⟵Rotate(probability=0.3,max_left_rotation=15,max_right_rotation=15)3:   P[i]⟵Zoom(probability=0.3,min_factor=1.1,max_factor=1.5)4:   P[i]⟵Flip_Left_Right(probability=0.5)5:   P[i]⟵Flip_Top_Bottom(probability=0.5)6:   P[i]⟵ Random_Distortion(probability=0.9,grid_width=2,grid_height=2,magnitude=8)7:**end for**

**Figure 2:**
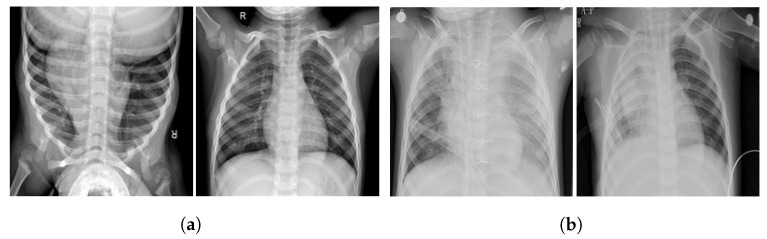
Samples of CXR images. (**a**) Normal and (**b**) pneumonia.

The purpose of the data augmentation is to balance the class distribution so as to improve the training process of the models, thereby enhancing their performance. A few sample X-ray images of normal and pneumonia classes are shown in Figure 2.

### 3.2. Data Splitting

The models are trained in order to discover and remove unnecessary patterns from the data. The dataset is divided into a training set and a testing set for this purpose. The training dataset is used to fit the model, and the algorithm makes predictions using the test set, which are then evaluated with the original labels to measure the method’s efficiency. We arbitrarily split the data into train and test halves with a proportion of 0.8 and 0.2, respectively, in this study. Table 2 indicates the number of training and testing samples.

### 3.3. Pre-Trained Deep Learning Models with Transfer Learning

In practice, building a deep network from the ground up is difficult. Weights are randomly allocated before training and changed repeatedly, relying on the datasets and the loss function in a large deep neural network. It takes time to change the weight iteratively, and accordingly, as a result of the paucity of training data, the deep network can also become overfit.

Transfer learning is a powerful tool for dealing with the mentioned deep learning problems [36]. It makes use of a pre-trained convolutional deep neural network that is developed on a different dataset [37]. A CNN can be trained to recognize hierarchical representations in images, as previously stated, and the information contained inside the weights of the pre-trained model may be used for different tasks. Lower-level features such as edges and vertices are collected at the lower-level convolutional layer. As a result, a new dataset must be used to train only higher-level representations, whereas representations at a lower level can also be transferred. The procedure of fine-tuning is to adjust the weights of higher hidden layers, and its effectiveness is determined by the distance between the origin and destination datasets. Many different tasks have been successfully employed to train a deep CNN using pre-trained weights, and the majority of well-known pre-trained models are trained using appropriate datasets.

#### 3.3.1. Convolutional Neural Network

The most important deep learning model is CNN, which uses convolution instead of general matrix multiplication. CNN is mostly used to classify objects based on image data [38,39]. A CNN is composed of three layers. The input layer creates an artificial input neuron that prepares the initial data for subsequent processing by the system. The hidden layers serve as a link between the input and output layers, with output layers producing results for the input layer.

**Figure 3:**
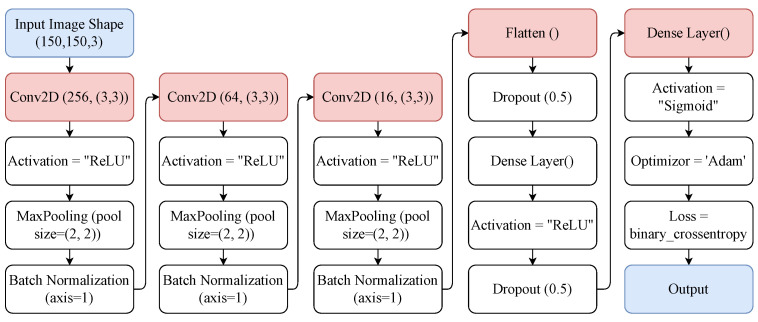
Architecture of CNN model used in this study.

Figure 3 shows the layer-wise architecture of the CNN model used in this study. Layers of CNN serve as the core building blocks of these models [40]. When a filter is applied to an input, the result is an activation. This is the fundamental process of convolution. After several iterations of the same filter to the same input, a feature map is constructed, which displays the locations and intensities of a detected pattern in the input, as well as an image of the pattern. A pooling layer is yet another component of a CNN architecture. The pooling layer helps to minimize the proportions of the feature sets. Therefore, the number of parameters to learn, as well as the amount of network processing, is reduced. The pooling layer adds the features in a feature map formed by a convolution layer in a specific area [40].

The CNN model consists of 18 layers: three 2D convolutional layers (2DConv), three max-pooling layers with 2 × 2 pool size, three batch normalization layers, four activation layers with a rectified linear unit (ReLU) activation function, two dropout layers with a 0.5 dropout rate, and two dense layers. The first layer of the CNN model consists of a 2DConv layer with 256 filters and a 3 × 3 kernel. This 2DConv layer is followed by the activation layer with the ReLU activation function. After this, we used a max-pooling layer with a 2 × 2 pool size. The batch normalization layer is used after the max-pooling layer, which is deployed with axis 1. There is again a 2DConv layer with 64 filters, and the last 2DConv layer consists of 16 filters. We used the binary_crossentropy loss function and Adam optimizer to compile the CNN models. In the end, we fitted the model with 25 epochs. Figure 3 shows the flow of the used CNN layers and the values of their parameters.

#### 3.3.2. Inception-V3 Model

The Inception-V3 model is an updated version of the Inception-V1 model. For greater model adaption, the Inception-V3 model uses a number of approaches to optimize the network [41]. It has a more extensive network than the Inception-V1 and V2 models. The Inception-V3 model is a deep CNN that is trained directly on a low-configuration computer. It is fairly difficult to train, and it takes a longer time, up to several days. This problem is solved via transfer learning [36], which keeps the last layer of the model for new categories. The parameters of the previous layers are kept, and the Inception-V3 model is deconstructed as the final layer is removed using the transfer learning technique.

#### 3.3.3. ResNet50 Model

ResNet50 is a CNN model with 50 layers. The ImageNet database which has been trained on over a million images provides a pre-trained version of the network that can be imported. Microsoft Research Asia suggested ResNet50 in late 2015 as a solution to the difficulties of training CNN models. As a result, creating a residual network with Keras for computer vision applications such as image classification is somewhat simplified [42].

#### 3.3.4. VGG-16 Model

The VGG-16 was the 2014 ILSVR (ImageNet) competition winner. It is widely regarded as the most sophisticated vision model design currently accessible. The ImageNet database was used to train the VGG-16 network. The number 16 in VGG-16 denotes the presence of 16 weighted layers [43]. The VGG-16 network provides good accuracy even if the image datasets are minimal because of its massive training. VGG-16 shows 92.7% accurate object detection and is a classification model capable of classifying 1000 images into 1000 unique categories. It is a common image classification algorithm that is simple to utilize with transfer learning. With batch normalization and the addition of new layers to neural networks, the training process may be sped up, making learning easier and the model more stable. We use dropout layers to avoid overfitting in the model, as well as the activation of the ReLu function and the Sigmoid in VGG-16. We used a 0.0001 learning rate with Adam optimizer and binary cross-entropy loss.

### 3.4. Ensemble Deep Learning

Ensemble learning involves training many models on the same dataset and making predictions using each of the trained models before integrating the predictions in some fashion to make the final prediction [44]. Ensemble models are sophisticated machine learning algorithms that increase the overall performance by incorporating decisions from different models [45]. Because deep neural networks are stochastic in nature, with each model learning certain filters and patterns, constructing ensembles of deep models is an excellent way to improve detection accuracy. We used three ensemble models in our study. Inception-V3 with CNN, ResNet50 with CNN, and VGG-16 with CNN are the three ensemble models used for training the X-ray images to increase the pneumonia detection accuracy.

**Figure 4:**
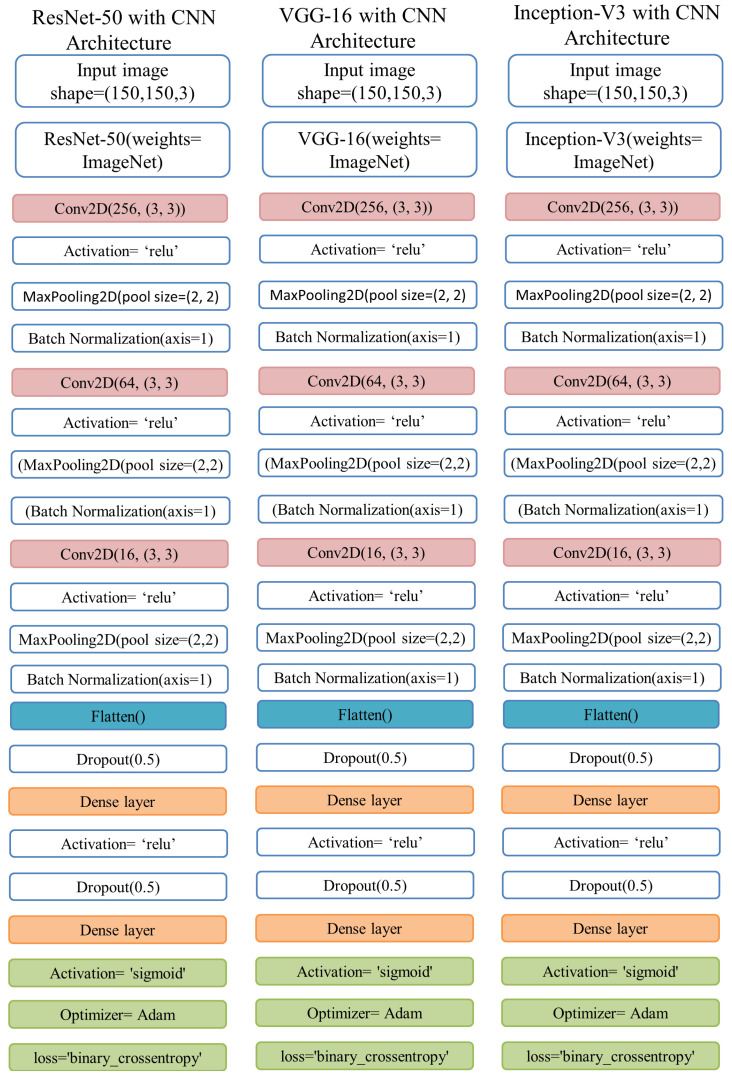
Architecture of three ensemble models used in this study.

For building an ensemble model, as shown in Figure 4, this study uses pre-trained deep learning models and a CNN model with input shapes (150, 150, 3). It is necessary to include both pre-trained models and the CNN model with the same input size in a sequential manner. The trainable parameters with their values are represented in Table 3. We froze dense layers of pre-trained models such as ResNet50 by using the include_top parameter with the value ‘False’. This parameter freezes the dense layers at the prediction level (fully connected layers), and we concatenate the CNN before these dense layers. Therefore, there is a difference in the trainable parameters of the individual ResNet50 and combined ensemble ResNet50 with CNN.

### 3.5. Evaluation Metrics

It is significant to learn how well the system is performing. For this purpose, various evaluation parameters are utilized. When the model classifies the data, it gives four possible outcomes, including true positive (TP), true negative (TN), false positive (FP), and false negative (FN). TP shows accurately predicted positive instances, TNs are correctly classified negative instances, while FPs and FNs are incorrectly classified as positive and negative instances, respectively. Recall, precision, accuracy, Cohen’s kappa, AUC, and F1 score are used as performance evaluation metrics in this study.

Accuracy is the number of correctly classified predictions by a model over the entire number of predicted instances and can be defined as

(1) $$Accuracy=\frac{TP+TN}{TP+FP+TN+FN}$$

Precision is the number of correct positive predictions from the total number of actual predictions classified by the model as positive and can be statistically defined as

(2) $$Precision=\frac{TP}{TP+FP}$$

The recall is the score of true positive predictions to the instances that actually belong to the positive class.

(3) $$Recall=\frac{TP}{TP+FN}$$

F1 score is an evaluation measure to estimate the model performance based on the average of precision and recall and is represented as:

(4) $$F1-score=2\times \frac{precision\times recall}{precision+recall}$$

Cohen’s kappa is also used to determine a classification model’s performance.

## 4. Results and Discussion

### 4.1. Experimental Setup

TensorFlow was used in an Anaconda Jupyter notebook to conduct the experiments. Using several CNN-based ensemble models, we investigated the accuracy of diagnosing pneumonia from chest X-ray images. The data were preprocessed and split into train and test sets in a 0.8 to 0.2 ratio, respectively. Fine-tuned deep learning models including ResNet50, Inception-V3, and VGG-16 were used to produce an ensemble with CNN. The ResNet50 with CNN, Inception-V3 with CNN, and VGG-16 with CNN are the three ensemble models that are used in this study.

### 4.2. Performance of Individual Deep Learning Models

The fine-tuned deep learning models, such as ResNet50, VGG-16, Inception-V3, and CNN, were utilized for experiments. All the models were trained using the binary cross-entropy loss function because our data contained normal and pneumonia classes, and for optimization, the Adam optimizer was used. The 64 batch size and 0.0001 learning rate were used for all models except for the ResNet50 model. We utilized a 0.001 learning rate and the SGD optimizer to optimize the ResNet50 model. A dropout layer was employed to prevent overfitting, and a batch normalization layer was implemented in the VGG-16, ResNet50, and CNN models to speed up model training, make learning simple, and minimize the error of generalization. All of the models were fitted with 25 epochs.

Table 4 contains the experimental results. CNN attained the highest accuracy as compared to other models. We observed that CNN performs well for detecting pneumonia, with an accuracy of 98.25%. This model attained 99.19% precision, 97.25% recall, and 98.21% F1 score. The Cohen’s kappa and AUC values were 96.51% and 98.24%. The Inception-V3 deep learning model scored 96.58% testing accuracy and 95.54% recall. The results indicate that the CNN model outperforms the other models in terms of accuracy.

The VGG-16 model attained 97.93% accuracy, 98.41% precision, 97.38% recall, and 97.89% F1 score. Cohen’s kappa was lower than other performance metrics, with 95.86%. The AUC of the VGG-16 model is 97.92%, which is higher than Inception-V3 and ResNet50 but low as compared to CNN. The ResNet50 model attained 97.87% testing accuracy, 98.15% precision, 97.51% recall, and 97.83% F1 score. The Cohen’s kappa was 95.74% and AUC was 97.87%. We observed that the CNN model attained the best accuracy among the four pre-trained deep-learning models, and Inception-V3 attained the lowest accuracy and Cohen’s kappa as compared to other models.

**Figure 5:**
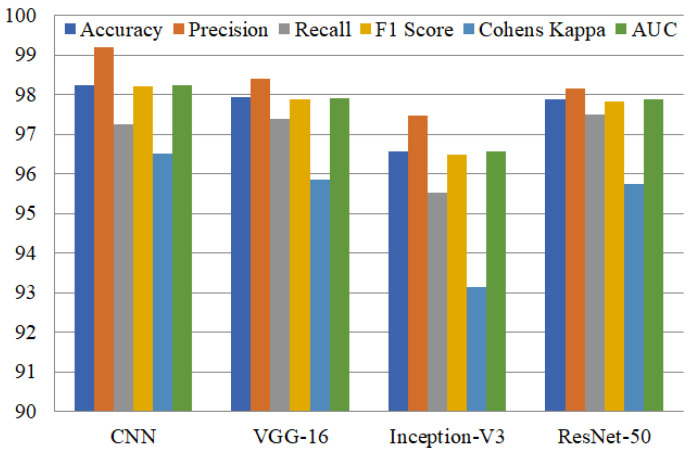
Comparison of individual models for performance evaluation metrics.

Figure 5 presents the results of fine-tuned deep learning models including CNN, VGG-16, ResNet50, and Inception-V3. We observe that CNN performs very well, with a precision score of 99.19% and Cohen’s kappa score for the Inception-V3 model of 93.15%, which is the smallest as compared to other models.

### 4.3. Training and Testing Results for Deep Learning Models

**Figure 6:**
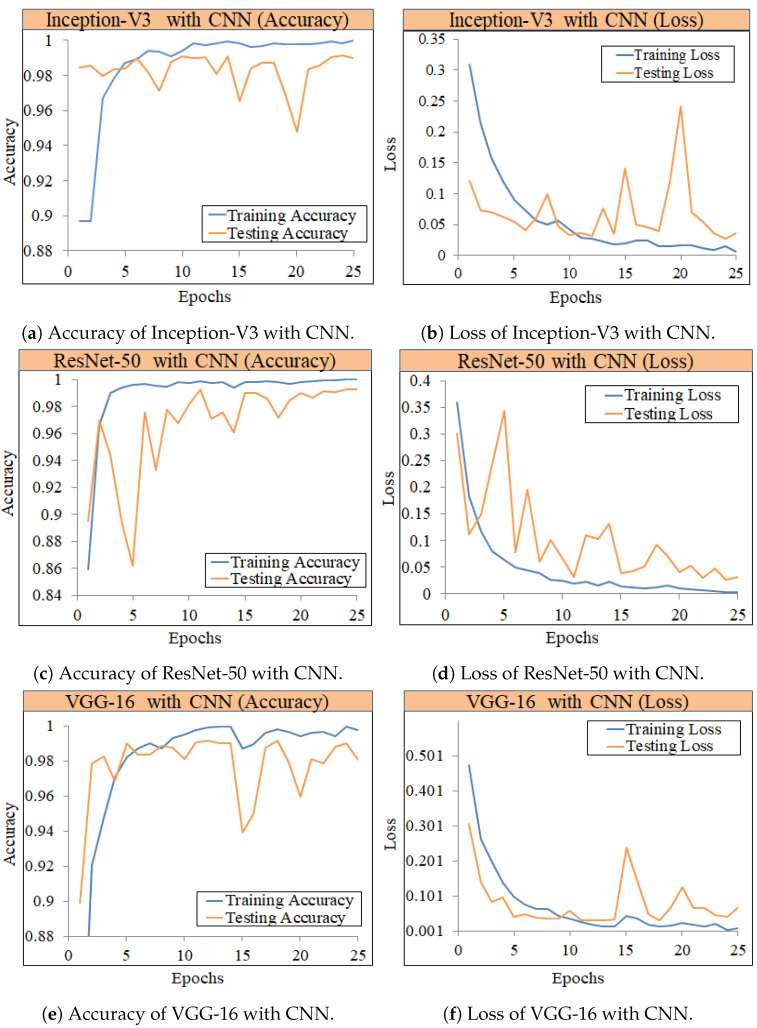
Training and testing accuracy of ensemble models. (**a**): Training and testing accuracy for Inception-V2 with CNN, (**b**): Training and testing loss for Inception-V2 with CNN, (**c**): Training and testing accuracy for ResNet-50 with CNN, (**d**): Training and testing loss for ResNet-50 with CNN, (**e**): Training and testing accuracy for VVG-16 with CNN and (**f**): Training and testing loss for VVG-16 with CNN.

The training and testing precision of the Inception-V3 with CNN model, as well as the loss curves, is depicted in Figure 6a. We can see that the accuracy of training data is 99.56% at epoch 13, and after this, variations are observed. Testing accuracy for the Inception-V3 with CNN increases with training data. We used a total of 25 epochs to train the ensemble deep learning models and attained superior performance with this number of epochs. At epoch 25, the training loss, as shown in Figure 6b, gradually dropped from 0.4322 to 0.0106, whereas the testing loss was reduced from 0.4987 to 0.0292. The ensemble deep learning model employs transfer weights with fine-tuning for identifying pneumonia from chest X-ray images. Using X-ray images, all of the models are trained and tested for normal and pneumonia classes.

Figure 6b,c show the training as well as testing accuracy and loss of the ResNet50 with CNN. We observe that the training data accuracy is 99.85% at epoch 13, and thereafter, we observed increases and decreases. We trained the ensemble deep learning model with a total of 25 epochs and achieved better performance with this number of epochs. The training loss decreased from 0.4140 to 0.0054 at epoch 25, and the testing loss decreased from 0.2873 to 0.0365. The training and testing accuracy of the VGG-16 with the CNN model, as well as the loss curves, is shown in Figure 6e,f. We can observe that the training data accuracy peaks at 99.66% at epoch 19, and thereafter, fluctuations are observed. The accuracy of the VGG-16 improves when more training data are added. We used a total of 25 epochs to train the model, and this number of epochs resulted in better performance. At epoch 25, the training loss dropped from 0.4742 to 0.0088, whereas the testing loss dropped from 0.3073 to 0.703.

### 4.4. Performance of Ensemble Deep Learning Models

Ensemble learning has been reported as highly influential in obtaining higher performance. Model ensembles are usually more accurate and error-tolerant than single models. The ensemble’s components must be characterized by accuracy and variety in their predictions for them to be effective. We achieved higher accuracy by combining VGG-16 with CNN, Inception-V3 with CNN, and ResNet50 with CNN compared to using single deep learning models. Inception-V3 with CNN attained the highest accuracy of 99.29% and precision of 98.83%, as shown in Table 5. This model obtained a recall of 99.73%, and an F1 score of 99.28%. The second-best ensemble model, ResNet50+CNN, achieves 99.09% accuracy, and the third-best ensemble model, VGG-16+CNN, attained 98.06% accuracy. Figure 6, shows the training and testing per epochs accuracies of deep learning models.

**Figure 7:**
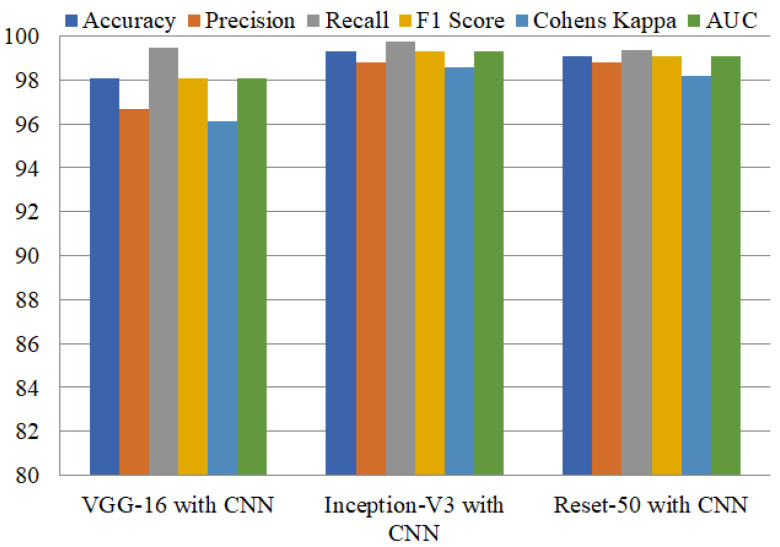
Performance comparison of ensemble models.

Performance comparison of all these models regarding the recall, precision, accuracy, F1 score, Cohen’s kappa, and AUC is shown in Figure 7. Results show the high performance of Inception-V3 with CNN, which performs exceptionally well, with 99.73% and 99.30% for accuracy and AUC, respectively.

### 4.5. Comparison with Existing Studies

To show the significance of the proposed approach, we carried out a performance comparison between the proposed approach and existing state-of-the-art approaches. Table 6 shows the comparison results with existing studies for pneumonia detection. We selected these studies for comparison regarding their use of the dataset. The selected studies used the same dataset for experiments and deployed an ensemble learning approach to detect pneumonia. For example, the study [22] employed transfer learning weights to reach 98.00% accuracy with a variety of CNN models. Similarly, the study [23] used an ensemble model, utilizing X-ray images to identify pneumonia, and achieved accuracy of 97.4%. In a similar fashion, ref. [26] used an ensemble deep learning model to identify COVID-19 and pneumonia from X-ray images and achieved significant accuracy of 95.05%. The study [28] also used an ensemble deep learning model to identify pneumonia and achieved an accuracy score of 98.81%. Performance comparison with these studies suggests that the current approach shows better performance for pneumonia detection from chest X-ray images, with 99.29% accuracy when combining Inception-V3 with CNN. Similarly, the performance of ResNet50 with CNN is also better than existing studies, with 99.08% accuracy.

### 4.6. K-Fold Cross-Validation Results

This study uses deep learning models to perform K-fold cross-validation in order to demonstrate the feasibility and efficiency of the models. Table 7 shows the results of 10-fold cross-validation. Results show that the models’ performance is also significant under K-fold cross-validation. The ensemble of Inception-V3 with CNN achieves the best accuracy score of 0.980 with the lowest standard deviation of 0.01. ResNet50 and the ensemble of ResNet50 with CNN also perform significantly better, with an accuracy score of 0.971 and standard deviations of 0.01 and 0.03, respectively. The VGG-16 combined with CNN produces an accuracy score of 0.984 with a standard deviation of 0.02. The 10-fold cross-validation results also show that the proposed approach is significant for pneumonia identification.

## 5. Conclusions

Pneumonia is a leading cause of death in infants and elderly people and takes hundreds of thousands of lives every year. Smokers, drinkers, surgery patients, asthma patients, persons with a compromised immune system, and those over 65 are at a higher risk of pneumonia. Early diagnosis of pneumonia and prompt treatment can potentially reduce the mortality rate of pneumonia. Pneumonia is often diagnosed by skilled specialists using a chest X-ray, but the lack of medical experts and the increase in pneumonia patients have recently introduced difficulties in its treatment. This study proposes an automatic pneumonia detection approach using chest X-ray images with deep learning-based ensemble models. Reduced training time and higher accuracy are obtained by leveraging the pre-trained models and combining them with a custom-built CNN model. The Inception-V3 ensemble achieved the highest accuracy among the models, with a 99.29% accuracy score, and 98.83%, 99.73%, and 99.28% scores for precision, recall, and F1, respectively. The ResNet50 ensemble obtained 98.93% accuracy, followed by the VGG-16 ensemble, with a 98.06% accuracy score. Results indicate that individual pre-trained models show poor performance as compared to their ensemble models.

## Figures and Tables

**Table 1:** Summary of the related work.

Ref.	Dataset	Training	Testing	Classifiers/Models	Year	Accuracy
[22]	Chest X-ray images (augmented)	9000	419	Different pre-trained CNN model	2020	0.98
[23]	Chest X-ray images	5232	624	Ensemble model	2020	97.4
[25]	Chest X-ray images	5216	640	CNN model	2021	90.78
[27]	Chest X-ray images (rearranged)	3722	2134	Proposed CNN model	2019	0.9373
[31]	COVID-19 X-ray images	90%	10%	Ensemble learning deep	2020	99
[24]	Chest X-ray images	80%	20%	Ensemble model	2021	86.3
[28]	Chest X-ray images (rearranged)	4686	1170	Ensemble deep learning model	2021	98.81
[26]	COVID-19 and CXR images joined dataset	6086		Ensemble deep learning model	2021	95.05
[29]	CT chest COVID-19 images dataset			Ensemble deep learning	2021	93.57
[30]	Heart disease dataset	800 records		Ensemble deep learning	2020	91

**Table 2:** Number of samples for train and test split.

Class	Training Set	Testing Set	Total
**Normal **	3089	786	3875
**Pneumonia**	3111	764	3875
**Total**	6200	1550	7750

**Table 3:** Trainable parameters for different deep learning models.

Model	Trainable Parameters
CNN	533,977
VGG-16	9,460,737
Inception-V3	38,537,217
ResNet50	262,401
Inception-V3 with CNN	26,645,113
ResNet50 with CNN	28,411,357
VGG-16 with CNN	16,052,505

**Table 4:** Results of fine-tuned pre-trained models.

Model	Accuracy	Precision	Recall	F1 Score	Cohen’s Kappa	ROC AUC
CNN	98.25	99.19	97.25	98.21	96.51	98.24
VGG-16	97.93	98.41	97.38	97.89	95.86	97.92
Inception-V3	96.58	97.46	95.54	96.49	93.15	96.57
ResNet50	97.87	98.15	97.51	97.83	95.74	97.87

**Table 5:** Results of ensemble models for pneumonia detection.

Models	Accuracy	Precision	Recall	F1 Score	Cohen’s Kappa	ROC AUC
VGG-16 + CNN	98.06	96.69	99.47	98.06	96.12	98.08
Inception-V3 + CNN	99.29	98.83	99.73	99.28	98.58	99.30
ResNet50 + CNN	99.09	98.82	99.34	99.08	98.19	99.10

**Table 6:** Performance comparison with other approaches.

Ref.	Techniques	Dataset	Accuracy (%)
[22]	Pre-trained CNN models	Chest X-ray	98.00
[23]	Ensemble model	Chest X-ray	97.4
[24]	Ensemble model	Chest X-ray	86.3
[25]	CNN model	Chest X-ray	90.78
[26]	Ensemble model	COVID-19, chest X-ray	95.05
[27]	CNN model	Chest X-ray	93.73
[28]	Ensemble model	Chest X-ray	98.81
This study	VGG with CNN	Chest X-ray	98.06
	Inception-V3 with CNN	Chest X-ray	99.29
	ResNet50 with CNN	Chest X-ray	99.08

**Table 7:** Results of 10-fold cross-validation for individual and ensemble models.

Models	Accuracy	Standard Deviation
CNN	0.963	+/− 0.05
ResNet50	0.971	+/−0.01
Inception-V3	0.960	+/−0.01
VGG-16	0.980	+/−0.00
ResNet50 with CNN	0.972	+/−0.03
Inception-V3 with CNN	0.981	+/−0.01
VGG-16 with CNN	0.984	+/−0.02

## Data Availability

Not applicable.
